# Negative cross-resistance between structurally different *Bacillus thuringiensis* toxins may favor resistance management of soybean looper in transgenic Bt cultivars

**DOI:** 10.1038/s41598-018-35965-5

**Published:** 2019-01-17

**Authors:** Nilson Rodrigues-Silva, Afonso F. Canuto, Diogo F. Oliveira, André F. Teixeira, Oscar F. Santos-Amaya, Marcelo C. Picanço, Eliseu J. G. Pereira

**Affiliations:** 1 Department of Entomology, Universidade Federal de Viçosa, Viçosa, MG 36570-900 Brazil; 2 National Institute of Science and Technology in Plant-Pest Interactions, Universidade Federal de Viçosa, Viçosa, MG 36570-900 Brazil; 3 Campus do Sertão, Universidade Federal de Sergipe, Nossa Senhora da Glória, SE 49680-000 Brazil

## Abstract

High adoption rates of single-gene *Bacillus thuringiensis* (Bt) Cry1Ac soybean impose selection pressure for resistance in the soybean looper, *Chrysodeixis includens*, a major defoliator in soybean and cotton crops. To anticipate and characterize resistance profiles that can evolve, soybean looper larvae collected from field crops in Brazil in 2013 were selected for resistance to Cry1Ac. Using two methods of selection viz., chronic exposure to Cry1Ac cotton leaves and the seven-day larval exposure to purified Cry1Ac on the artificial diet, 31 and 127-fold resistance was obtained in 11 and 6 generations of selection, respectively. The resistance trait had realized heritability of 0.66 and 0.72, respectively, indicating that most of the phenotypic variation in Cry1Ac susceptibility of the soybean looper larvae was due to additive genetic variation. The Cry1Ac-selected populations showed positive cross-resistance to Cry1Ab (6.7–8.7 fold), likely because these Bt toxins have a very similar molecular structure. Importantly, the Cry1Ac-selected populations became more susceptible to Cry2Aa and Cry1Fa, showing negative cross-resistance (up to 6-fold, *P* < 0.05). These results indicate that Cry1Ac, Cry1Fa, and Cry2A are compatible in a multi-toxin approach to minimize the risk of rapid adaptation of the soybean looper to Bt toxins.

## Introduction

Plant biotechnology has helped in the development of pest-resistant cultivars, which are increasingly important in the face of growing pressure to reduce broadcast pesticides typically used in crop protection^1^. Transgenic crops producing *Bacillus thuringiensis* (Bt) insecticidal toxins have been extensively used as a major tool for controlling insect pests worldwide^2–4^. In two decades of use, these crops have been reported to provide substantial agronomic, environmental, economic, and societal benefits^5,6^. Nevertheless, the sustainable use of these crops is threatened by the rapid evolution of resistance^7,8^.

Historically, Bt cultivars of corn (*Zea mays*) and cotton (*Gossypium* sp.) were commercially introduced prior to those of soybean (*Glycine max*)^2,9^. While Bt cotton and corn expressing more than one toxin were launched in the 2000s^4^, single-trait Bt soybean was introduced a decade later^10–12^, although new multi-trait Bt soybean are in the process of commercial release^13,14^. The single-toxin Bt soybean produces Cry1Ac, which is also produced in some Bt cotton technologies, such as Bollgard (Cry1Ac) and Bollgard II (Cry1Ac + Cry2Ab), and all are simultaneously deployed in some countries^9,11,15^. The Bt Cry1Ac soybeans were first commercialized in 2013 in South America^12^, which accounts for more than half of the global production^16^. Soybean cultivars carrying this Bt technology have been extensively adopted in Brazil, Argentina, Paraguay, and Uruguay^2^, targeting the main Lepidoptera pests like the velvetbean caterpillar, *Anticarsia gemmatalis* (Erebidae) and the soybean looper, *Chrysodeixis includens* (Noctuidae), in spite of not reaching the high-dose condition for the latter^10^.

The soybean looper is a notorious pest not only in soybean but also in cotton, where a relatively high number of larvae can survive and reproduce on Bt cotton foliage producing Cry1Ac^17,18^. In such a less-than-high-dose scenario, even though some Bt susceptible larvae may recover from sub-lethal exposure to the toxin and transmit susceptibility alleles to subsequent generations^19^, some carriers of resistance alleles may pass the resistance allele to the next generation and increase the rate of Bt resistance evolution faster than expected from current resistance management modeling^20,21^. Bt soybean grown predominantly over 60% of 39.1 million ha and Bt cotton occupying 70% of 1.2 million ha^2^, both producing the same Cry1Ac toxin, exert tremendous selection pressure on common pests to evolve resistance. This is particularly the case for the soybean looper, which is relatively less susceptible to Cry1 toxins and biopesticides^10,22,23^. The risk of Bt resistance in the soybean looper is further increased because the larvae typically have sheltered feeding habits within the plant canopy, which is challenging for effective insecticidal sprays that may be needed for integrated pest/resistance management.

In order to manage pest adaptation (often referred to as insect resistance management) to Bt crops, the primary strategy has been to ensure that effective refuges of non-Bt host plants occur near Bt crops. Ideally, these crops must have toxicity that is high enough to render resistance recessive (i.e., to kill nearly all insects heterozygous for Bt resistance), although for some pests the low innate susceptibility may make it difficult to reach a high-dose condition^21,24^. Other approaches that can be used with refuges are “pyramids”, which are plants that produce two or more Bt toxins effective against the same pest^21,25–28^, and alternating with new toxins for which no resistance is reported^24^. Structurally distinct toxins can be effective for resistance management when pest populations are susceptible to all toxins deployed in the Bt crop^24,28^ and there is negligible cross-resistance among them in order to meet the redundant-killing principle^21,26^. Also appropriate would be if individuals that have alleles conferring resistance to a single-toxin Bt crop are hypersusceptible to another toxin, in which case there would be negative cross-resistance between the two toxins^29^.

Resistant populations of insects targeted by Bt crops provide an opportunity to assess the conditions that favor the success of resistance management strategies, including the genetic basis of the resistance^30,31^, cross-resistance^7,32,33^, fitness costs^34^, and frequency of resistance alleles in the field^35,36^. Selection experiments that generate resistance similar to field resistance^31,37,38^ are useful in assessing the risk of resistance evolution^39–43^. Such efforts help to elucidate the mode of action of Bt toxins, assess the risk of field-level resistance and develop resistance management strategies^30,44^. Here, we report on the ability of the soybean looper to respond to selection for resistance to Cry1Ac in the laboratory and its respective realized heritability. Importantly, we also report negative cross resistance in the Cry1Ac selected soybean looper to Cry2Aa and Cry1F, which may have important implications for resistance management in Bt crops, especially soybean.

## Results

### Response to selection for Cry1Ac resistance

**Figure 1:**
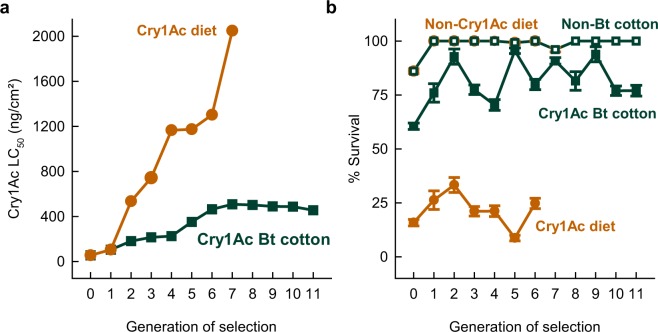
Response to selection for resistance to the Cry1Ac *Bacillus thuringiensis* (Bt) toxin in larvae of the soybean looper as affected by two methods of selection, namely chronic exposure to Cry1Ac cotton leaves and the seven-day larval exposure to purified Cry1Ac on the artificial diet. (**a**) Increase in the median lethal concentration (LC_50_) for larvae of the individuals selected in the previous generation. (**b**) Larval survival rates (±SE) for the selected individuals (i.e., survivors on Bt Cry1Ac cotton or diet overlaid with Cry1Ac) as compared to those reared on plain food (i.e., non-Bt isoline cotton or artificial diet).

Estimates of median lethal concentration (LC_50_) values and survival rates over the generations of selection (Fig. 1) show that soybean looper larvae increased the resistance to Cry1Ac when exposed to the Bt cotton leaf tissues throughout larval development, or to the toxin on the surface of the artificial diet for seven days of exposure. Selection with Cry1Ac overlaid on the surface of the diet resulted in higher resistance in each generation of selection (Fig. 1a), as it imposed a greater intensity of selection than did the Bt cotton leaf tissues producing Cry1Ac (Fig. 1b: mean selected proportion of 27% and 81%, respectively). In fact, with only two generations of selection using the purified toxin, the increase in the LC_50_ values was similar to that achieved in six generations of selection using the Bt cotton leaves (Fig. 1a).

### Realized heritability

Realized heritability (*h*^2^) estimates were 0.66 and 0.72 for the respective Cry1Ac and Bt-cotton selected populations, and the corresponding number of generations needed for a 10-fold increase in LC_50_ values were ca 3 and 8 generations (Table 1). After eleven generations of selection with leaf tissues of Bt cotton, Cry1Ac LC_50_ values increased from 19 to 503 ng/cm^2^ and the slope of the probit line increased from 1.04 to 2.34, while after six generations of selection with Cry1Ac, toxin LC_50_ values increased from 19 to 2048 ng/cm^2^ and the slope increased from 1.04 to 2.11 (Table 1).

**Table 1:** Estimation of realized heritability (*h*^2^) and number of generations to a 10-fold increase in resistance to the Cry1Ac *Bacillus thuringiensis* toxin in two populations of soybean looper selected using chronic exposure to Cry1Ac Bt cotton leaves (BG1-Sel) or seven-day larval exposure to purified Cry1Ac on the artificial diet (1Ac-Sel).

Population of soybean looper	Number of gene- rations selected	Estimate of mean response per generation			Estimate of mean selection differential per generation							
		Initial LC_50_ (log)	Final LC_50_ (log)	Response to selection (*R*)	Mean Percent survival after selection	Intensity of selection^a^	Initial slope	Final slope	Phenotypic standard deviation	Selection differential (*S*)	Realized heritability (*h*²)	Number of generations to a 10-fold increase in resistance
BG1-Sel	11	1.283	2.701	0.129	81	0.33	1.04	2.49	0.59	0.22	0.66	8
1Ac-Sel	6	1.283	3.311	0.338	27	1.23	1.04	2.38	0.59	0.82	0.72	3

LC_50_, lethal concentration of Cry1Ac needed to kill 50% of the larvae exposed to the toxin. ^a^Intensity of selection was calculated according to Tabashnik and Falconer and Mackay^50,78^.

### Level of resistance and cross-resistance

As indicated by the ratios between the LC_50_ values for the selected and unselected larvae (Table 2), selection with Cry1Ac cotton leaves produced a 31-fold resistance in 11 generations of selection, while exposure to purified Cry1Ac produced a 127-fold resistance to Cry1Ac in only 6 generations of selection (Table 2). Cry1Ab LC_50_ values increased 7–9 fold in both selected insect populations, indicating positive cross-resistance between Cry1Ab and Cry1Ac in the soybean looper (Table 2). In contrast, for larvae selected with Cry1Ac cotton leaves, there was a significant reduction in Cry2Aa and Cry1Fa LC_50_ values of 4 and 6-fold, respectively (*P* < 0.05, based on the likelihood ratio test and non-overlapping fiducial limits); likewise, there was a 6-fold decrease in Cry2Aa LC_50_ value for the population selected with purified Cry1Ac toxin. These results indicate negative cross-resistance between Cry1Ac and Cry2Aa or Cry1Fa in the soybean looper, even if a conservartive criterion is used (i.e., lack of overlap between fiducial limits of the LC_50_ values for unselected and selected strains) (Table 2).

**Table 2:** Resistance and cross-resistance to *Bacillus thuringiensis* (Bt) toxins in two soybean looper populations, one selected using chronic exposure to Cry1Ac Bt cotton leaves (BG1-Sel) and the other using seven-day larval exposure to purified Cry1Ac on the artificial diet (1Ac-Sel). The bioassays were conducted during the last generation of selection.

Toxin	Population	Slope ± SE	LC_50_ (95% fiducial limits)^a^ ng/cm²	Resistance ratio^b^ (95% confidence limits)	*χ* ^2 c^	*P*	N^d^
Cry1Ac	Bt-Unsel	1.85 ± 0.14	16.1 (13.22–19.71)	1	2.66	0.752	545
	BG1-Sel	2.34 ± 0.27	502.82 (398.51–636.50)	31.10 (22.82–42.35)	1.27	0.937	236
	1Ac-Sel	2.11 ± 0.23	2048.4 (1567.8–2893.4)	126.67 (94.10–170.51)	5.43	0.365	437
Cry1Ab	Bt-Unsel	3.39 ± 0.64	86.40 (64.25–107.27)	1	2.21	0.697	230
	BG1-Sel	1.25 ± 0.16	576.90 (358.90–1763.00)	6.70 (4.11–10.83)	9.02	0.108	359
	1Ac-Sel	0.76 ± 0.10	748.00 (304.96–3863.40)	8.66 (3.95–18.99)	6.67	0.246	301
Cry1Fa	Bt-Unsel	5.43 ± 0.81	117. (101.25–135.88)	1	2.24	0.691	256
	BG1-Sel	2.77 ± 0.30	22.12 (18.25–25.84)	0.20 (0.16–0.23)	0.97	0.965	539
Cry2Aa	Bt-Unsel	4.03 ± 0.68	24.30 (19.51–30.60)	1	0.23	0.998	248
	BG1-Sel	3.92 ± 0.54	6.13 (5.15–7.32)	0.25 (0.19–0.33)	3.28	0.657	252
	1Ac-Sel	2.52 ± 0.31	3.99 (2.50–5.80)	0.17 (0.12–0.28)	10.36	0.065	443

^a^LC_50_, (Lethal Concentration causing 50% mortality, in ng/cm^2^) was estimated by probit analysis using Polo-Plus^79^.

^b^Resistance ratio = LC_50_ selected population/LC_50_ for control population, indicates the level of resistance or cross-resistance, that is, how many times the selected population is less susceptible than the control, unselected population to a particular toxin; values in parentheses represent the 95% confidence limits for the resistance ratio^79^.

^c^Chi-square statistic with its *P* value for df = 5.

^d^Number of insects tested in the bioassays.

### Survival assays on soybean leaf tissue

**Figure 2:**
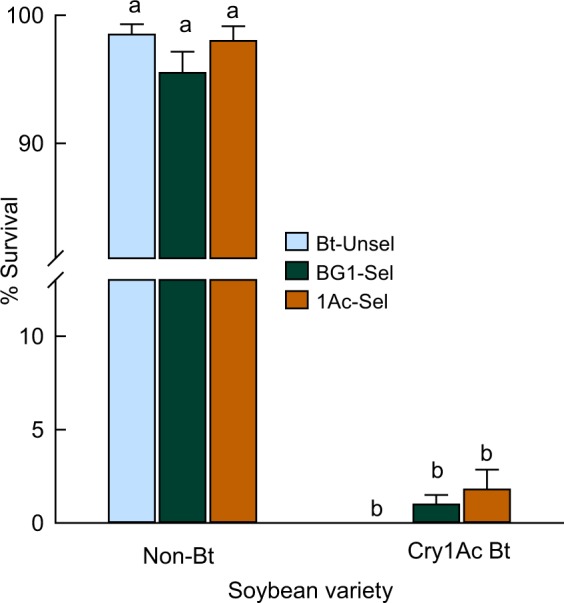
Testing whether soybean looper larvae from the Cry1Ac-selected (BG1-Sel and 1Ac-Sel) and unselected populations (Bt-Unsel) survive on foliage of Cry1Ac Bt soybean. Shown are mean (±SE) 3-day survival rates for neonates (n = 200) released on foliage excised from Cry1Ac-producing and near-isoline soybean plants. Columns with same letter are not significantly different (α = 0.05, Fisher’s LSD procedure after ANOVA).

Despite the 127-fold resistance to Cry1Ac in the 1Ac-Sel soybean looper population, their larvae did not survive on Cry1Ac soybean foliage (Fig. 2). The was no significant difference in larval survival of the three insect populations on either non-Bt or Bt soybean (*F*_2, 110_ = 1.37, *P* = 0.32; *F*_2, 109_ = 0.01, *P* = 0.99). However, overall insect survival differed significantly between Bt and non-Bt soybean (*F*_1, 110_ = 13545.8, *P* < 0.01) (Fig. 2). Most larvae of any selected or unselected populations died in 3 days on Bt soybean. On non-Bt soybean, the survival rates were similar for both selected and unselected control populations (Fig. 2), indicating no fitness cost of resistance to early-instar larval survival.

## Discussion

Field-derived larvae of the soybean looper responded to selection for resistance to Cry1Ac in the laboratory, generating up to 127-fold resistance to the toxin, which is consistent with other selection experiments using purified Bt toxin^30,45–48^ and plant leaf tissue producing Bt toxin^7,31,38,39,49^. These results indicate that either method of selection (i.e., leaf tissues of Bt plants or larval diet containing Bt toxin) can be used to produce resistant insect populations, which are important tools for anticipating the risk assessment of resistance development in field settings^7,30,47^.

Whereas using purified Bt toxin on the larval diet may allow better control of the intensity of selection and avoid confounding effects of plant allelochemicals, using the Bt plant foliage may better represent the larval exposure under field settings^50^, selecting for a more realistic resistance profile. Gossypol interaction with Cry1Ac in Bt cotton may have affected the ability of soybean looper to evolve resistance as fast as with pure Cry1Ac^51^. In addition, Cry1Ac protein levels may have varied in the cotton foliage^52,53^, even though we meticulously grew cotton plants in optimized soil conditions and standardized the growth stage and the leaf age used in the experiment. To our knowledge, this is the first report of selection of Bt resistant populations of soybean looper. Importantly, despite the 127-fold resistance to Cry1Ac in the 1Ac-Sel soybean looper population, their larvae did not survive on Cry1Ac soybean foliage. The low survival of 1Ac-Sel larvae on Cry1Ac soybean foliage may be due to the relatively high titer of Bt toxin^10,54,55^ or its synergism with soybean allelochemicals^56^.

The higher level of resistance reached by the 1Ac-Sel population indicates that the rate of resistance evolution to Cry1Ac in soybean looper is linked to the intensity of the selection^57^. The realized heritability (*h*^2^) values for Cry1Ac resistance were quite high (0.66 and 0.72), which means that most of the phenotypic variation for Cry1Ac resistance in the selected soybean looper populations is due to genetic variation^50^, and indicates that the soybean looper has high potential to develop resistance to Cry1Ac. The higher heritability value for the selection using Cry1Ac on artificial diet is indicative of a greater contribution of genes as compared to that for selection using Bt cotton leaves. The latter reflects that low concentration of Cry1Ac and presence of allelochemicals. The slow resistance evolution in the soybean looper using Bt cotton may reflect a likely scenario for field-evolved resistance in the near future in a soybean agroecosystem, particularly if Bt soybean cultivars carry natural resistance to the looper and/or expresses high Bt toxin content.

Despite trying to obtain resistant strains representative of those found in the field by mimicking field conditions in our laboratory selection experiment^50,57^ we cannot guarantee that the resistance is exactly like that which may evolve in the field^58^, because the conditions will differ under field settings. Nevertheless, laboratory selection experiments have often produced resistance similar to field-evolved resistance^8,31,34,36,37,59^. Our results indicate that the pre-existing Cry1Ac resistance alleles^12^ may not be so rare in the field (i.e., >0.001), such that the risk of populations of soybean looper to evolve field resistance to Cry1Ac soybean should be further investigated.

Selection for Cry1Ac resistance resulted in different levels of cross-resistance to other Cry toxins regardless of the method of toxin exposure. Importantly, our estimated LC_50_ values are comparable to those reported for soybean loopers in Brazil^60^ and the United States^61^. This latter study also reported that Cry1Ac and Cry1Fa toxins have specific binding sites on the midgut brush border membrane vesicles of soybean looper larvae, although the toxins share some, but not all, binding sites in the insect midgut^61^. Here, Cry1Ac and Cry1Ab showed low but significant (i.e., 6.7–8.7 fold) positive cross-resistance, which is not surprising given the likely resistance mechanism^62^ and the similarity in the amino acid sequences of domains II and III (i.e., 99% and 51%, respectively)^32^. Despite a paucity of published reports on competition binding assays between Cry1Ac and Cry1Ab in the soybean looper, these toxins do share binding sites^63,64^ or show positive cross-resistance^33,65^ in other Lepidoptera.

In addition, there was no positive cross-resistance between Cry1Ac and Cry1Fa or Cry2Aa in the two selected soybean looper populations, supporting an absence of common binding sites for these pairs of toxins on brush border membrane of the midgut based on competitive and specific binding studies^61^. Interestingly, our data indicate that the resistance to Cry1Ac negatively correlates with resistance to Cry1Fa or Cry2Aa (i.e., the Cry1Ac-selected soybean loopers became more susceptible to Cry1Fa and Cry2Aa). This is one of the few empirical evidences for negative cross-resistance or negatively correlated resistance involving Bt toxins (reviewed in^29^), deserving investigation of its genetic basis (i.e., if caused by a single or different genes^29^), which in this case may encode altered receptor proteins interacting with these Bt toxins in their intoxication route^66^. In practical terms, negative cross-resistance may be exploited for resistance management^29^; for example, Cry1F and Cry2A toxins may be used to preferentially kill soybean loopers that are resistant to Cry1Ac.

Although some variation exists in the patterns of cross-resistance between Cry1A and Cry2A^47,67–69^, these toxins seem to be compatible for resistance management in most pest species. Here, the clear absence of positive cross-resistance to Cry1Fa and Cry2Aa provides empirical evidence that these toxins do not share binding sites in receptor proteins^61,70^. These findings are relevant because Cry2A or Cry1F toxins are pyramided with Cry1Ac in second-generation Bt cotton^71,72^ and soybean^13,14^ in the Americas. Most importantly, our results indicate that Cry2A and/or Cry1F are compatible with Cry1Ac in a multi-toxin approach for resistance management of soybean looper. This is critical in the stewardship and optimal management strategy^24^ of new Bt soybean varieties^13,14^ that are to be introduced in the market for controlling soybean looper and other lepidopteran pests.

## Material and Methods

### Insect collection and rearing

Field populations of soybean looper (ca. 500 third to fifth instar larvae) were collected from non-Bt soybean (*G. max*) and dry beans (*Phaseolus vulgaris*) on farms of the Federal University of Viçosa, in Viçosa and Coimbra counties, Minas Gerais state, Brazil, in April 2014. The larvae were brought to the laboratory and reared individually on leaves of the respective host crops (i.e., non-Bt soybean or dry beans). Two to three batches of pupae (80♂ + 80♀) were placed in two polyvinylchloride cages, 20 cm diameter × 30 cm height, lined internally with sulfite paper as substrate for oviposition. Adults were fed a 10% honey solution in distilled water, and eggs were collected daily and stored in an incubator until hatching. Neonates were reared at 27 ± 1 °C, 70 ± 10% r.h. with 16:8 (light:dark) cycle on artificial diet^73^. In the F_1_ generation, the larvae were divided in three subpopulations; one was selected with purified Cry1Ac toxin (1Ac-Sel), another was selected with Cry1Ac cotton leaf tissue (BG1-Sel), and a third subpopulation was left unselected (Bt-Unsel) and maintained on artificial diet using methods described above, keeping the population size at approximately 200 adults per generation.

### Source of cotton for selection and soybean for leaf-bioassays

Transgenic plants producing Cry1Ac used were Bollgard cotton (event MON 531, Monsanto do Brasil, São Paulo, SP) and Intacta soybean (event MON 87701 x MON 89788, Monsanto do Brasil, São Paulo, SP). Isoline or near isoline non-Bt cotton (Delta OPAL, Monsanto do Brasil, São Paulo, SP) and non-Bt soybean (MSOY 8866, Monsanto do Brasil, São Paulo, SP) were used as controls. To obtain appropriate leaves for the bioassays, cotton and soybean plants were cultivated in the greenhouse following standard cultivation practices^74,75^. Cotton was sown every three months in 15-L pots with substrate composed of 3 parts of soil, 2 parts cattle manure, and 2 parts of sand to obtain plants with normal levels of Bt protein expression^52,53,76^. The plants were irrigated twice or three times a day depending on soil moisture conditions, and leaves were collected from cotton plants 45–50 d after emergence. Soybean plants were field-grown using cultivation practices as recommended for the crop, and leaves were excised when plants were in the R2-R4 growth stages^74^. Soil fertilization was as recommended for cotton^75^ or soybean crops^74^. The plants were inspected weekly for mechanical pest control or disposal of infested plants when needed. Immunodetection assays using ImmunoStrip STX 74500 kit (Agdia Inc., Elkhart, IN) were used according to the manufacturer’s instructions to confirm the presence or absence of the Cry1Ac trait in the Bt or non-Bt isoline plants from which foliage was excised.

### Bt toxins and bioassays

The Cry1A toxins (Cry1Ab, Cry1Ac and Cry1Fa) and Cry2Aa protoxin used in the experiments were obtained from Dr. Marianne P. Carey (Case Western Reserve University, OH). The proteins were purified on HPLC, shipped as lyophilized powder, and stored at −80 °C until use, when fresh dilutions were prepared as follows. Cry1 toxins were solubilized in 100 mM Na_2_CO_3_ buffer (pH 10.3, containing 10 mM DTT) and Cry2A protoxin in 50 mM Na_2_CO_3_ buffer (pH 12.1, containing 5 mM EDTA and 10 mM EGTA) to produce the stock concentration (1 mg/ml) for each Bt toxin. These were further diluted with 0.1% Triton-X 100 to obtain the appropriate concentrations used in the bioassays.

All bioassays used in the selection experiment and cross-resistance study were repeated twice and included at least seven different concentrations of purified Cry toxin plus a control (i.e., 0.1% Triton-X 100 only) applied to the diet surface^77^. A single neonate (<24 h after hatching) was placed in each well of a 128 well-tray (CD International, Pitman, NJ), and held for seven days at 27 ± 1 °C, 24 h scotophase, and 80% r.h. until assessment of larval mortality^45,77^. Larvae of the Cry1Ac-selected population were not bioassayed against purified Cry1Fa as they were low in availability when the assays were conducted.

### Selection experiment using purified Cry1Ac toxin

This experiment was conducted in parallel with that using Bt cotton leaf sections. Methods were adapted from Gould *et al*.^47^ and Pereira *et al*.^45^. Initial bioassays to determine the population susceptibility to Cry1Ac were conducted as previously described, using the same toxin source that was later used in the selection experiment. Bioassays were done using graded Cry1Ac concentrations applied on the surface of artificial diet^77^. Larval mortality was recorded after seven days of exposure and analyzed by probit regression to determine the concentration that kills 90% of the larvae (LC_90_), which was used as the concentration applied on the diet surface for the next generation of selection. The following Cry1Ac concentrations were used from the first to sixth generation of selection, respectively: 215, 535, 1568, 1868, 3004, and 4058 ng/cm^2^ (Table S1). At least 2,500 neonates were exposed to Cry1Ac every generation of selection, and after seven days of feeding on the toxin-treated diet, the larvae with size (i.e., weight) similar to those of the control were selected. These larvae were transferred to the untreated artificial diet and reared until pupation. The adults were held in mating cages as previously described. A portion of the offspring from the parents selected in the previous generation was bioassayed as above to estimate the gain by selection. This process was repeated during six generations of selection.

### Selection experiment using Cry1Ac cotton foliage

A chronic selection experiment using Cry1Ac cotton leaves throughout larval development was started in the first generation of the field-collected laboratory colony in 2014. In each generation of selection, we transferred 160 batches of 10 neonates to 16-well-plastic trays (Advento do Brasil, Diadema, São Paulo) (10 larvae/well), each well containing a cotton leaf section. After three days, we assessed the number of survivors, and they were transferred to new 16-well trays (3 larvae/well). From then on, cotton leaves were replaced every three days until pupation, when the insects were transferred to mating cages and held as previously described. The selection experiment took place during 11 generations. Soybean looper larvae were also reared in parallel on non-Bt cotton leaves to estimate natural mortality in the absence of selection. In each generation of selection, the gain by selection (i.e., increase in resistance) was estimated using bioassays with purified Cry1Ac protein as described above.

### Leaf tissue assay using Cry1Ac soybean

We tested the hypothesis that the Cry1Ac resistance developed in the laboratory-selected soybean looper populations would be high enough to allow for survival on leaf tissues of the Cry1Ac Bt soybean. In a completely randomized experiment with 20 replications, we exposed larvae of the two selected and the control populations to foliage of Bt soybean (Intacta) and its non-Bt near isoline (MSOY8866, Monsanto do Brasil, São Paulo, SP). Soybean foliage was excised in the R2-R4 growth stages, quickly placed in buckets with water, brought to the laboratory, thoroughly rinsed with distilled water, and placed on paper towels to dry for 15 min at room temperature. The Cry1Ac or control soybean foliage was cut into 3-cm^2^ pieces and placed in 50-ml plastic cups. Batches of 10 neonates (<1 day old) were transferred to each cup and maintained as described previously. After three days, we recorded the number of survivors and calculated the survival rate for each experimental unit (i.e., each cup, total n = 120). The total number of larvae tested in the bioassay was 1200 (400 per insect population).

### Realized heritability

Following Falconer and Mackay^78^ and Tabashnik^50^, we calculated the realized heritability (*h*^2^) as *h*^2^ = *Response to selection* ÷ *Selection differential*. In this equation, the response to selection (*R*) was calculated as: *R* = [Log (final LC_50_)−Log (initial LC_50_)]/*n*, where the final LC_50_ is the LC_50_ of the population after six generation of selection with purified toxin or Bt cotton foliage, respectively; the initial LC_50_ is the LC_50_ of the base parental population before selection, and *n* is the number of generations selected with Bt cotton or the purified toxin. The selection differential was calculated as follows: *Selection differential* = *i* *×* *σ*_*p*_, where *i* is intensity of selection calculated according to Falconer and Mackay^78^, and *σ*_*p*_ is the phenotypic standard deviation, which was calculated as follows: *σ*_*p*_ = [0.5 × (initial slope + final slope)]^−1^. Finally, the number of generations required for a 10-fold increase in LC_50_ (*G*) was calculated as: *G* = 1/*R*.

### Statistical analyses

The probit model was fit to bioassay data using Polo-Plus software^79^ to estimate the concentration causing 50% mortality (LC_50_) and their 95% fiducial limits as well as the slope of the concentration-response lines and their standard errors. The data were adjusted for natural mortality relative to controls when needed. Lethal concentration ratios (i.e., resistance or cross-resistance ratios) and their respective 95% confidence limits^79^ were determined using the unselected control population as reference for comparison, and considered significantly different (*P* < 0.05) when they did not include the value one. The significance of cross-resistance between toxins was also assessed by the failure of 95% fiducial limits at LC_50_ estimates to overlap, which is quite conservative^79,80^. The data from leaf tissue assays were analyzed using a two-way analysis of variance; the two main effects were insect population and plant phenotype (Bt or non-Bt soybean), both considered fixed factors. Means were separated using a comparison wise error rate (α) of 0.05^81^.

## Electronic supplementary material

Table S1

## Data Availability

The datasets generated and/or analyzed during the current study are available from the corresponding author on reasonable request.
