# Supplementary material for: Negative cross-resistance between structurally different Bacillus thuringiensis toxins may favor resistance management of soybean looper in transgenic Bt cultivars
Source: Sci Rep. 2019 Jan 17;9:199. doi: 10.1038/s41598-018-35965-5 (PMC6336840; doi:10.1038/s41598-018-35965-5)
Supplement: Supplementary file 1 — Table S1 [file 41598_2018_35965_MOESM1_ESM.pdf]

Negative cross-resistance between structurally different *Bacillus thuringiensis* toxins may favor resistance management of soybean looper in transgenic Bt cultivars

Nilson Rodrigues-Silva[1,2,3], Afonso F. Canuto[1], Diogo F. Oliveira[1], André F. Teixeira[1], Oscar F. Santos-Amaya[1], Marcelo C. Picanço[1] & Eliseu J. G. Pereira[1,2]

[1]Department of Entomology, and [2]National Institute of Science and Technology in Plant-Pest Interactions, Universidade Federal de Viçosa, Viçosa, MG, 36570-900, Brazil. [3]Campus do Sertão, Universidade Federal de Sergipe, Nossa Senhora da Glória, SE, 49680-000, Brazil.

Supplementary information

Table S1. Summary of the susceptibly profile obtained in the diet-surface Cry1Ac bioassays against larvae of a soybean looper a population selected for resistance to Cry1Ac using the Bt toxin on the artificial diet.

Generation of selection	Slope ± SE	Cry1Ac LC_{90} (95% FL)	χ^2	*P*
1	2.15 ± 0.19	215.7 (176.7 - 276.8)	4.73	0.450
2	1.82 ± 0.21	535.0 (288.6 - 1768.1)	7.70	0.173
3	2.74 ± 0.32	1568.4 (1180.3 - 2378.5)	1.33	0.931
4	3.20 ± 0.41	1868.2 (1410.6 - 2711.7)	1.21	0.943
5	3.12 ± 0.42	3003.6 (2276.3 - 4469.0)	1.85	0.763
6	2.38 ± 0.27	4057.9 (2457.5 - 10716.0)	8.52	0.130
7	Nd	> 2000	Nd	Nd

In the column head, LC_{90} is the lethal concentration (ng/cm^{2}) that causes 90% mortality of the population and its 95% fiducial limits, estimated through probit analysis using Polo-Plus. χ^2 is the chi-square statistic with its *P* value for the goodness-of-fit to the probit model. Nd, not determined due to insufficient concentration-response (i.e., the range toxin concentration tested, up to 2000 ng/cm^2, did not cause mortality to estimate the regression line).
